# The Biological Effects of Bilirubin Photoisomers

**DOI:** 10.1371/journal.pone.0148126

**Published:** 2016-02-01

**Authors:** Jana Jasprova, Matteo Dal Ben, Eleonora Vianello, Iryna Goncharova, Marie Urbanova, Karolina Vyroubalova, Silvia Gazzin, Claudio Tiribelli, Martin Sticha, Marcela Cerna, Libor Vitek

**Affiliations:** 1 Institute of Medical Biochemistry and Laboratory Diagnostics, 1st Faculty of Medicine, Charles University in Prague, Prague, Czech Republic; 2 Italian Liver Foundation, CSF, Trieste, Italy; 3 Institute of Chemical Technology Prague, Prague, Czech Republic; 4 Faculty of Science, Charles University in Prague, Prague, Czech Republic; 5 The Institute for Mother and Child, Prague, Czech Republic; 6 4th Department of Internal Medicine, 1st Faculty of Medicine, Charles University in Prague, Prague, Czech Republic; Kermanshah University of Medical Sciences, ISLAMIC REPUBLIC OF IRAN

## Abstract

Although phototherapy was introduced as early as 1950’s, the potential biological effects of bilirubin photoisomers (PI) generated during phototherapy remain unclear. The aim of our study was to isolate bilirubin PI in their pure forms and to assess their biological effects *in vitro*. The three major bilirubin PI (ZE- and EZ-bilirubin and Z-lumirubin) were prepared by photo-irradiation of unconjugated bilirubin. The individual photoproducts were chromatographically separated (TLC, HPLC), and their identities verified by mass spectrometry. The role of Z-lumirubin (the principle bilirubin PI) on the dissociation of bilirubin from albumin was tested by several methods: peroxidase, fluorescence quenching, and circular dichroism. The biological effects of major bilirubin PI (cell viability, expression of selected genes, cell cycle progression) were tested on the SH-SY5Y human neuroblastoma cell line. Lumirubin was found to have a binding site on human serum albumin, in the subdomain IB (or at a close distance to it); and thus, different from that of bilirubin. Its binding constant to albumin was much lower when compared with bilirubin, and lumirubin did not affect the level of unbound bilirubin (Bf). Compared to unconjugated bilirubin, bilirubin PI did not have any effect on either SH-SY5Y cell viability, the expression of genes involved in bilirubin metabolism or cell cycle progression, nor in modulation of the cell cycle phase. The principle bilirubin PI do not interfere with bilirubin albumin binding, and do not exert any toxic effect on human neuroblastoma cells.

## Introduction

**Fig 1:**
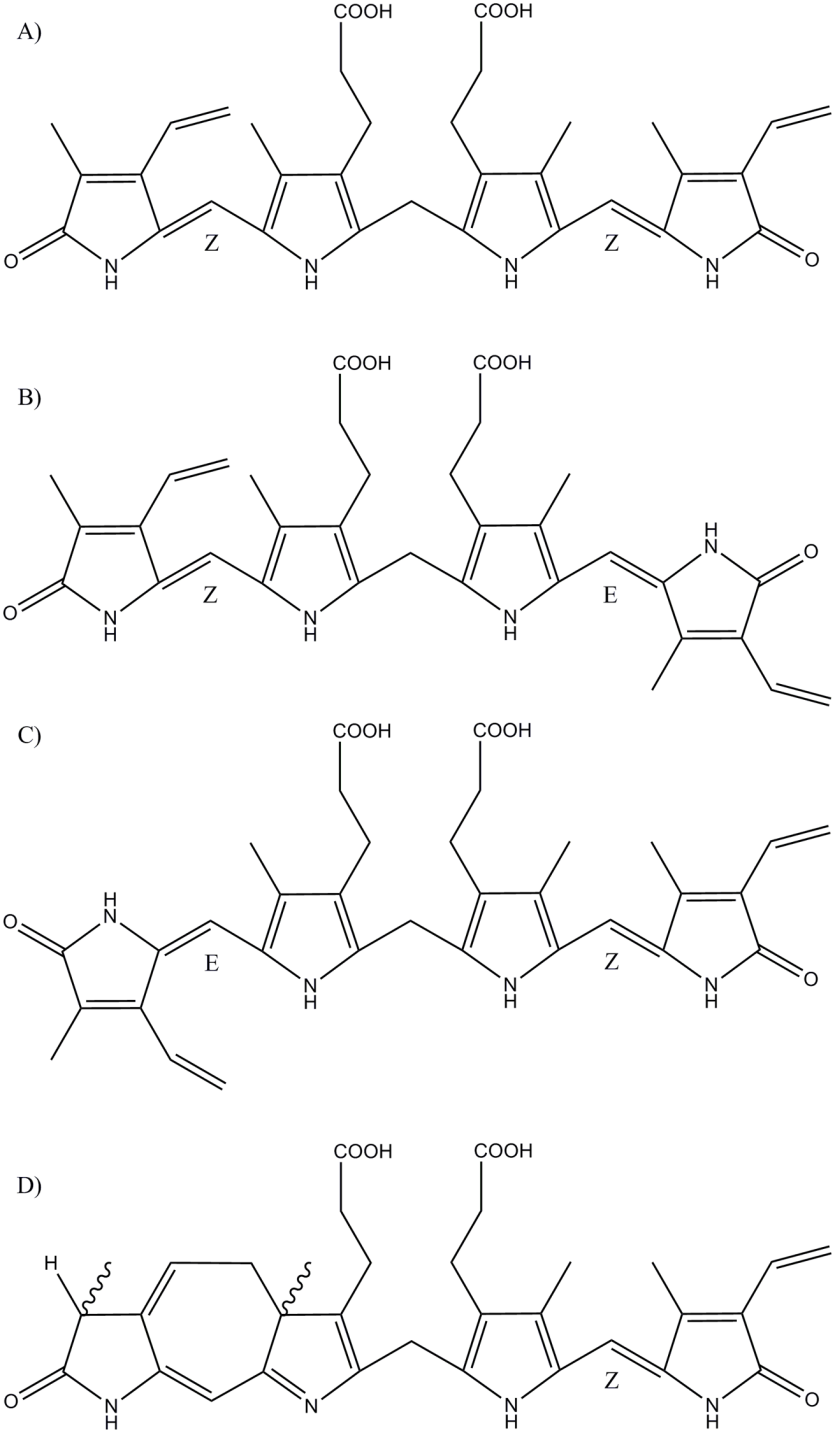
Bilirubin and its photoderivatives. (A) Z,Z-Bilirubin IXα. (B) Z,E-Bilirubin IXα. (C) E,Z-Bilirubin IXα. (D) Z-Lumirubin IXα.

Phototherapy as a treatment option for neonatal hyperbilirubinemia was first used by Cremer and co-workers in the 1950's [1]. This technique is based on the fact that blue-green light converts bilirubin into more polar derivatives. Configurational and structural photoisomers (PI), ZE- and EZ-bilirubins, lumirubin (also called cyclobilirubin) (Fig 1) [2–4], as well as propentdyopents and other oxidation products [5,6], can be relatively easily excreted from the body.

Although phototherapy for neonatal hyperbilirubinemia is accepted as the 'gold standard' of treatment, it may be accompanied with side effects such as impairment of thermoregulation, mineral dysbalance [7], and direct genotoxic effects on lymphocyte DNA [8]. This might also be one of the reasons for the increased prevalence of allergic conditions reported in these newborns [9]. In addition, intensive phototherapy in very low birth-weight newborns has been associated with increased risk of ileus [10]; also surprisingly by increased mortality, as demonstrated in the Collaborative Phototherapy Trial, as well as the NICHD Neonatal Network Trial [11,12].

The neurotoxicity of bilirubin is directly associated with the concentration of the fraction unbound to albumin (or other solubilizing substances), which is called Bf (bilirubin free) [13,14]. Bf is critically dependent on the presence of compounds that are potentially competing with bilirubin in binding to albumin [15]. Nothing is known about whether bilirubin PI may affect the bilirubin-albumin interaction. Previous studies on the biological effects of bilirubin photoproducts [16–23] suffered from a major limitation (insufficient purity of the bilirubin photoproducts), as well as inconsistent study designs. Thus, there is still uncertainty on the potential toxicity of bilirubin breakdown products not only for the central nervous system, but also other organs.

Therefore, the aim of the current study was to isolate and characterize pure forms of bilirubin PI; and then to assess their potential effects on bilirubin-albumin binding, as well as their possible biological effects *in vitro* using the neuroblastoma cell line SH-SY5Y.

## Materials and Methods

### Chemicals

The bilirubin (AppliChem, Darmstadt, Germany) was purified before use, according to McDonagh and Assissi [24]. The chloroform and di-n-octylamine were purchased from Sigma (MO, USA). The methanol was from Merck (Darmstadt, Germany), and ammonia from Penta (Czech Republic).

Because of light-sensitivity of bilirubin and bilirubin PI, all procedures were carried out under dim light in aluminium wrapped flasks. Evaporation was performed under vacuum and stream of nitrogen.

### Preparation of PI

The pure bilirubin photoderivatives were prepared as previously described [25–27] with a slight modification of the original protocol. Briefly, bilirubin (100 mg) was dissolved in slightly basified methanol (1% NH_3_ solution in methanol); the solution underwent 90 minutes of photo-irradiation using a Lilly phototherapeutical device (TSE, Czech Republic), composed of a field of LEDs emitting light (wavelength range = 430–500 nm with a broad peak between 445 and 474 nm (width at half max) with a maximal spectral irradiance of 100 μW/cm^2^/nm corresponding to the total irradiance of 3.1 mW/cm^2^. The sample was then evaporated under vacuum, dissolved in pure methanol, decanted from the residual bilirubin, and re-evaporated. The residue of bilirubin PI was protected from light, and stored at -20°C until use.

### Thin layer chromatography

**Fig 2:**
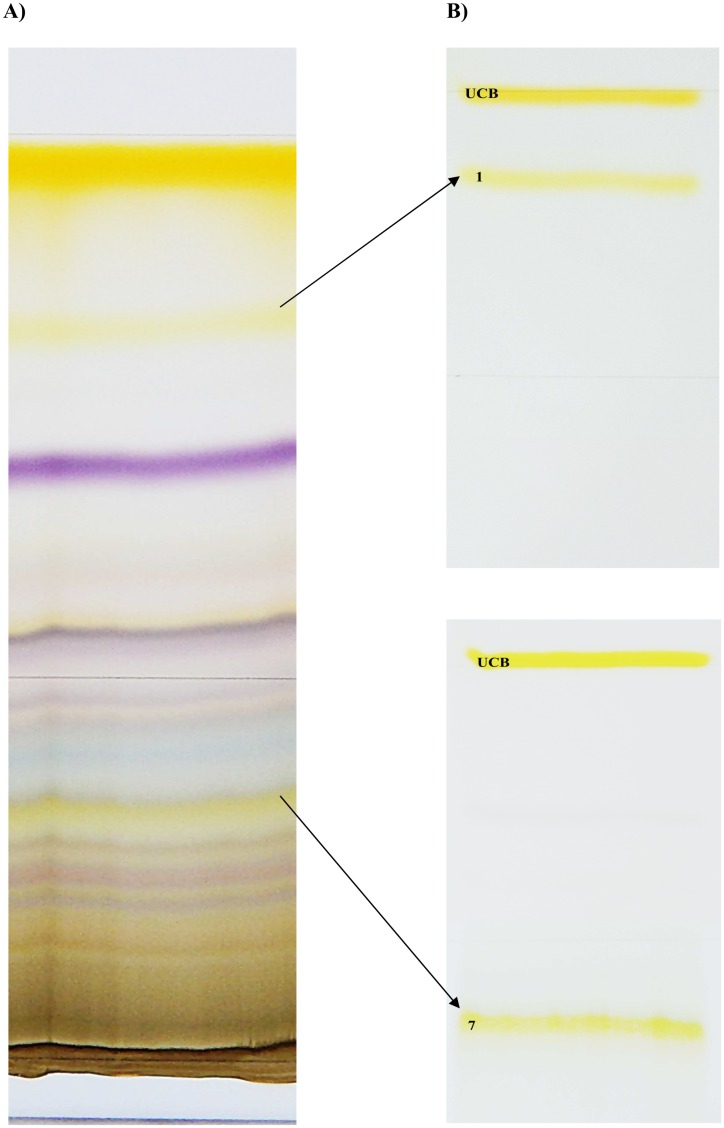
Compounds produced by bilirubin phototherapy. (A) TLC plate after first chromatography. (B) Most important compounds 1 and 7 were separated by re-chromatography from the 1^st^ (upper panel), and 7^th^ zone (lower panel). UCB, unconjugated bilirubin.

**Fig 3:**
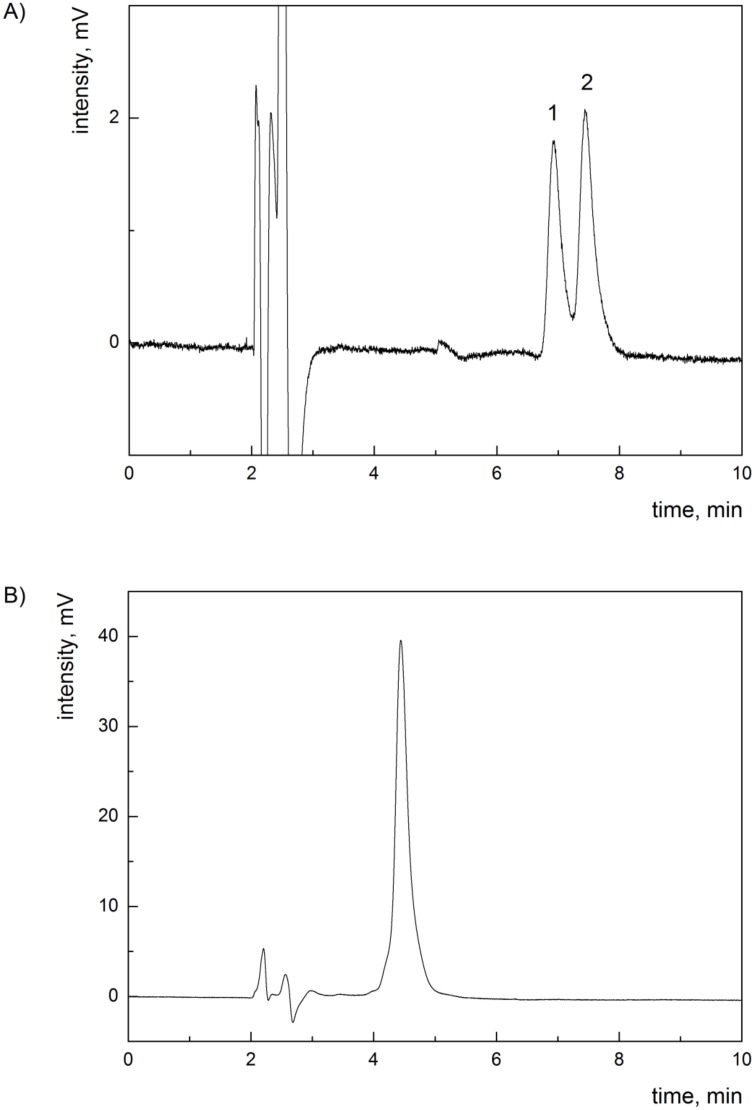
HPLC chromatograms of isolated bilirubin PI. (A) HPLC chromatogram of band 1 from TLC—mixture of ZE/EZ-bilirubins; peak 1 = EZ-bilirubin, peak 2 ZE-bilirubin. (B) HPLC chromatogram of band 7 from TLC—Z-lumirubin.

The residue after photo-irradiation was dissolved in a small amount of methanol:chloroform (1:1, v/v), and separated by thin layer chromatography (200 x 200 x 0.25 mm Kieselgel 60 TLC plates [Merck, Darmstadt, Germany]; the mobile phase = chloroform:methanol:water, 40:9:1, v/v/v). During the first chromatography, the mixture of bilirubin derivatives was separated into 8 major bands, which were extracted using the mobile phase, evaporated to dryness, and then re-chromatographed using the same conditions. The individual separated compounds were re-extracted, the solvent evaporated, and the isolated compounds were stored at -20°C until used. The isolated compounds 1 and 7, corresponding to ZE/EZ-bilirubins and lumirubin, as verified by HPLC [28,29] (Figs 2 and 3, also see Results), in a 1:1 ratio, were used for functional and biological studies as being representative of the principle bilirubin PI.

### High-performance liquid chromatography analyses

The HPLC analyses were performed using an Agilent 1200 system (CA, USA) with a diode-array detector. The method was a modification of that by McDonagh *et al*. [28,29]. The mobile phase consisted of 0.1 M di-n-octylamine acetate in methanol and water; the stationary phase was represented by a Poroshell 120, SB-C18 column (4.6 x 100 mm, 2.7 μm; Agilent, CA, USA). Samples were prepared by mixing 20 μl of bilirubin solution with 180 μl of ice-cold 0.1 M di-n-octylamine acetate in methanol, then vortexed and centrifuged to eliminate proteins. Twenty μl of the prepared sample was injected onto the column.

### Spectrophotometry

The absorption spectra of pure bilirubin PI were measured using a Lambda 25 spectrophotometer (Perkin Elmer, USA) in the spectral range from 200 to 900 nm. Samples for analyses were diluted in pure methanol, and measured against methanol as the blank.

### Mass Spectrometry

Mass spectra were measured by using an Escuire 3000 mass spectrometer (Bruker Daltonics, Germany) coupled with electrospray ionization. All samples for MS analysis, dissolved in methanol, were injected directly on MS and measured in a negative mode. The masses were scanned in the range between 50 and 800 m/z. The capillary exit was set at -106.7 V.

### Estimation of binding constant by fluorescence quenching

A fluorescence quenching method was used for measurement of either lumirubin-albumin or bilirubin-albumin interactions, as well as their binding constants [30]. The determination is based on the fact that bile pigments do not emit any fluorescence; on the other hand, human serum albumin (HSA) contains a tryptophan residue (Trp-214) in the subdomain IIA, which is responsible for its fluorescence. Thus, the binding constant for bilirubin and lumirubin to HSA was determined by quenching of the intrinsic Trp fluorescence.

For the *Ka* determination, formula (1) was used:

$$Ka=\frac{F_{0}-F}{F\left(C_{L}-n\frac{F_{0}-F}{F_{0}}C_{p}\right)}$$ (1)

- where *F*_*0*_ was the fluorescence of HSA without a quencher, *F* the fluorescence of HSA with a quencher, *C*_*L*_ was the quencher concentration, and *C*_*P*_ was the concentration of HSA.

The effect of cooperative binding of lumirubin and bilirubin was also studied, and the results were compared to the *Ka* obtained in the systems with biliverdin and gossypol, which served as a displacing agent of bilirubin from HSA [31,32].

### Characterization of the bile pigment albumin binding sites by circular dichroism (CD) spectroscopy

Unbound pigments were dissolved in 0.1 mol/L NaOH and mixed with the HSA solution in PBS (pH 7.4) at the molar ratio [pigment]/[HSA] = 1/1, the concentration of the pigment was 1.5 x 10^−5^ mol/L. Bilirubin did not undergo aggregation, as verified by spectrophotometry [33]. CD spectra were obtained using a J-810 spectropolarimeter (Jasco, Japan) and analyzed as described elsewhere [34]. The method is based on the fact that the unbound pigment does not give any CD signal, and monitoring of their CD intensity provides information about their co-binding or displacement and localization in the albumin subdomains. For determination of the subdomain for lumirubin binding two compounds were used, hemin and bilirubin, as marker ligands for subdomain IB and IIA, respectively [31,35].

### Determination of Bf

The effect of bilirubin PI on Bf levels was studied by a peroxidase method [36]. Briefly, the standard stock solution of horseradish peroxidase (HRP) was made (1 mg/ml), which was diluted by PBS to different concentrations ranging from 1:2 to 1:100. For each enzyme dilution the K_p_ value (oxidation constant of bilirubin) was determined. For enzyme standardization (K_p_ constant determination, also see below), the solution of bilirubin in PBS without albumin was used (bilirubin concentrations were between 1 and 3 μM). Bilirubin absorbance was measured at 440 nm (Beckman Coulter DU-730 spectrophotometer, CA, USA). Afterwards, 5 μl of H_2_O_2_ and 10 μl of HRP were added, the solution was slightly mixed, and the decrease of absorbance at 440 nm in 60 s was measured. The *K*_*p*_ constant was counted according to *K*_*p*_ calculation formula (2):

$$K_{p}=\frac{V_{0}}{\left[Bf\right]\cdot \left[HRP\right]}$$ (2)

- where *K*_*p*_ = constant for oxidation of bilirubin, and *V*_*0*_ = the initial oxidation velocity (expressed as ΔAbs/min).

The measurements of Bf in PBS-containing albumin or in complete culture medium was performed with enzymes whose *K*_*p*_ values were similar (in our experimental setup, enzyme dilutions of 1:2, 1:3, and 1:4 were used). We first determined the bilirubin concentration corresponding (under the conditions used) to 140 nM (approximately 24 μM bilirubin). Then, we studied whether Bf is affected by the addition of increasing concentrations of bilirubin PI (15% and 30% of total bilirubin concentrations, respectively, based on the fact that as much as 30% decrease of total serum/plasma bilirubin concentrations can be achieved during phototherapy of neonatal jaundice [37]). A mixture of ZE/EZ-bilirubins and Z-lumirubin was used for these studies. To assess the possible effect of solvent on Bf concentration, DMSO was used in the same concentration as for dissolving of bilirubin PI. To check the stability of bilirubin PI, concentrations of bilirubin IXα, and EZ/ZE-bilirubins as well as Z-lumirubin after 1 and 24 hrs in the incubation medium were analysed by HPLC method (see above). Whereas all studied pigments did not change their concentrations after 1 hr and bilirubin IXα, and EZ/ZE-bilirubins were stable also after 24 hrs under conditions used, concentrations of Z-lumirubin decreased to 31% after 24 hrs.

### Cell culture studies

The SH-SY5Y human neuroblastoma cell line was used for the *in vitro* studies (ATCC, Manassas, VA, USA). Authentication of the cell line was confirmed by independent laboratory (Generi Biotech, Czech Republic). The cells were tested for Mycoplasma contamination using the MycoAlert luminescence test (Lonza, Switzerland). Cells were cultured in a mixture of MEM Eagle and Ham’s F12 media (1:1, v/v), containing 15% of fetal bovine serum, in 75 cm^2^ culture flasks, at 37°C, in a 5% CO_2_ atmosphere. For functional tests, cells were seeded at a concentration of 50,000 cells per 1 cm^2^.

### Cell viability analyses

The effect of bilirubin (24 μM), pure bilirubin PI (5%, 15%, and 30% of bilirubin PI in complete culture media), and the combination of bilirubin with bilirubin PI on cell viability was analyzed by both MTT (Sigma, Germany) as well as by luminescent CellTiter-Glo (Promega, USA) tests, using a Sunrise Microplate Reader (Tecan, Austria) and Synergy 2 Multi-mode Microplate Reader (BioTek, USA), respectively.

### Gene expression studies

The effect of bilirubin and bilirubin PI (24 μM bilirubin (corresponding to 140 nM Bf), 15% bilirubin PI, and a mixture of bilirubin and 15% PI) on the expression of genes involved in heme catabolism and in the regulation of the cell cycle (Table 1) was investigated in SH-SY5Y cells exposed to pigments for 1 and 24 hrs, respectively. Briefly, total RNA was isolated in TriReagent (Sigma-Aldrich, St Louis, MO, USA) according to the manufacture's protocol and stored at -80°C until analysis. RNA quantity and purity were evaluated spectrophotometrically at 260 nm, and RNA integrity was evaluated by agarose gel electrophoresis. Retrotranscription of total RNA (1 μg) was performed with an iScript cDNA Synthesis Kit (Bio-Rad Laboratories, Hercules, CA, USA) according to the manufacturer's instructions. The final cDNA was conserved at -20°C until used. The primers for the targeted genes and the two housekeeping genes [hypoxanthine-guanine phosphoribosyltransferase (Hprt1) and glyceraldehydes 3-phosphate dehydrogenase (Gapdh)] were designed using Beacon Designer 2.0 software (PREMIER Biosoft International, Palo Alto, CA, USA). The quantitative analysis of gene expression was performed by real-time PCR. The reaction was performed on 25 ng of cDNA, with the corresponding gene-specific sense and anti-sense primers (250 nM, all genes) with iQ SYBER Green Supermix in an I-Cycler iQ thermocycler (Bio-Rad Laboratories, Hercules, CA, USA). The thermal cycler conditions consisted of 3 min at 95°C; plus 40 cycles each at 95°C for 20 s, 60°C for 20 s, and 72°C for 30 s. A melting curve analysis was performed to assess product specificity. The relative quantification was made using iCycler iQ software, version 3.1 (Bio-Rad Laboratories, Hercules, CA, USA), by the ΔΔCt method, taking into account the efficiencies of the individual's genes, and normalizing the results to the two housekeeping genes [38,39]. The levels of mRNA were expressed relative to a reference sample. The results are expressed as the mean ± SD.

**Table 1:** List of genes used for gene expression analyses.

Gene	Accession Number	Forward	Reverse	Ampl Lenght	Efficiency
**CFTR/MRP (ABCC1)**	NM_004996.3	tgatggaggctgacaagg	gcggacacatggttacac	127	99.20
**MDR/TAP (ABCB1)**	NM_000927	tgctcagacaggatgtgagttg	aattacagcaagcctggaacc	122	92.90
**HMOX1**	NM_002133.2	atgccccaggatttgtca	cccttctgaaagttcctcat	95	95.00
**HMOX2**	NM_001127204.1	tgagtataacatgcagatattca	ccatcctccaaggtctct	75	92.40
**BLVRA**	NM_000712	cgttcctgaacctgattg	aaagagcatcctccaaag	87	96.00
**CyclinD1**	XM_006718653.1	acagatgtgaagttcatt	tagtaggacaggaagttg	110	96.50
**CyclinE1**	NM_001238.2	agcccttgggacaataatg	cggtcatcatcttctttg	76	94.50
**GAPDH**	NM_002046	tcagccgcatcttcttttg	gcaacaatatccactttaccag	146	103.00
**HPRT1**	NM_000194	acatctggagtcctattgacatcg	ccgcccaaagggaactgatag	193	105.00

HPRT1 and GAPDH were used as house-keeping genes.

### Heme oxygenase activity determination

The activity of heme oxygenase (HMOX) was measured as described previously [40]. In brief, culture media harvested from SH-SY5Y batches were added to CO-free, septum-sealed vials. CO released into the vial headspace was quantified using gas chromatography with a reduction gas analyzer (Peak Laboratories, Mountain View, CA, USA).

### Flow cytometry

Cells for flow cytometry analyses were treated with bilirubin (24 μM corresponding to 140 nM Bf), bilirubin PI (15%), and a combination of bilirubin and PI for 1 and 24 hrs. At the end of the treatment the medium was aspired, the cells were washed twice with PBS, then fixed by adding 5 ml of cold (-20°C) 80% ethanol drop-wise under constant gentle vortexing. After centrifugation (310 x g; RT; 6 min), the sediments were re-suspended in 1 ml of staining solution in PBS containing 0.1% v/v Triton X-100, 20 μg/ml propidium iodide (PI), and 0.2 mg/ml DNAse free RNaseA. Samples were incubated in the dark for 30 min at RT, and subjected to FACS analysis (cytometer BD FACSCalibur TM; and CellQuest software, BD Biosciences, San Jose, CA). Data were collected for 10,000 events per sample.

### Statistical analyses

Data are presented as the median and 25–75% range. Differences between variables were evaluated by the Mann-Whitney Rank Sum test. Differences were considered statistically significant when p-values were less than 0.05. Statistical analyses were performed using Prism 6 software (GraphPad, CA, USA).

## Results

### The isolation of bilirubin PI

Photo-exposure of unconjugated bilirubin, under the conditions defined above, lead to the generation of 18 different PI (Fig 2). These compounds were further characterized by HPLC, UV/VIS spectrophotometry, and mass spectrometry to check for their purity and identity.

Out of these 18 individual substances, several were clearly identified (unconjugated bilirubin, biliverdin, ZE/EZ-bilirubin, and lumirubin); the others are likely to represent unstable, oxidized, and as yet undefined intermediates. Pigments 1 and 7 (Fig 2), identified as ZE/EZ-bilirubins and lumirubin (the principal bilirubin PI), were used for further functional studies. (Figs 2 and 3).

### Effect of bilirubin PI on the bilirubin-albumin binding

**Fig 4:**
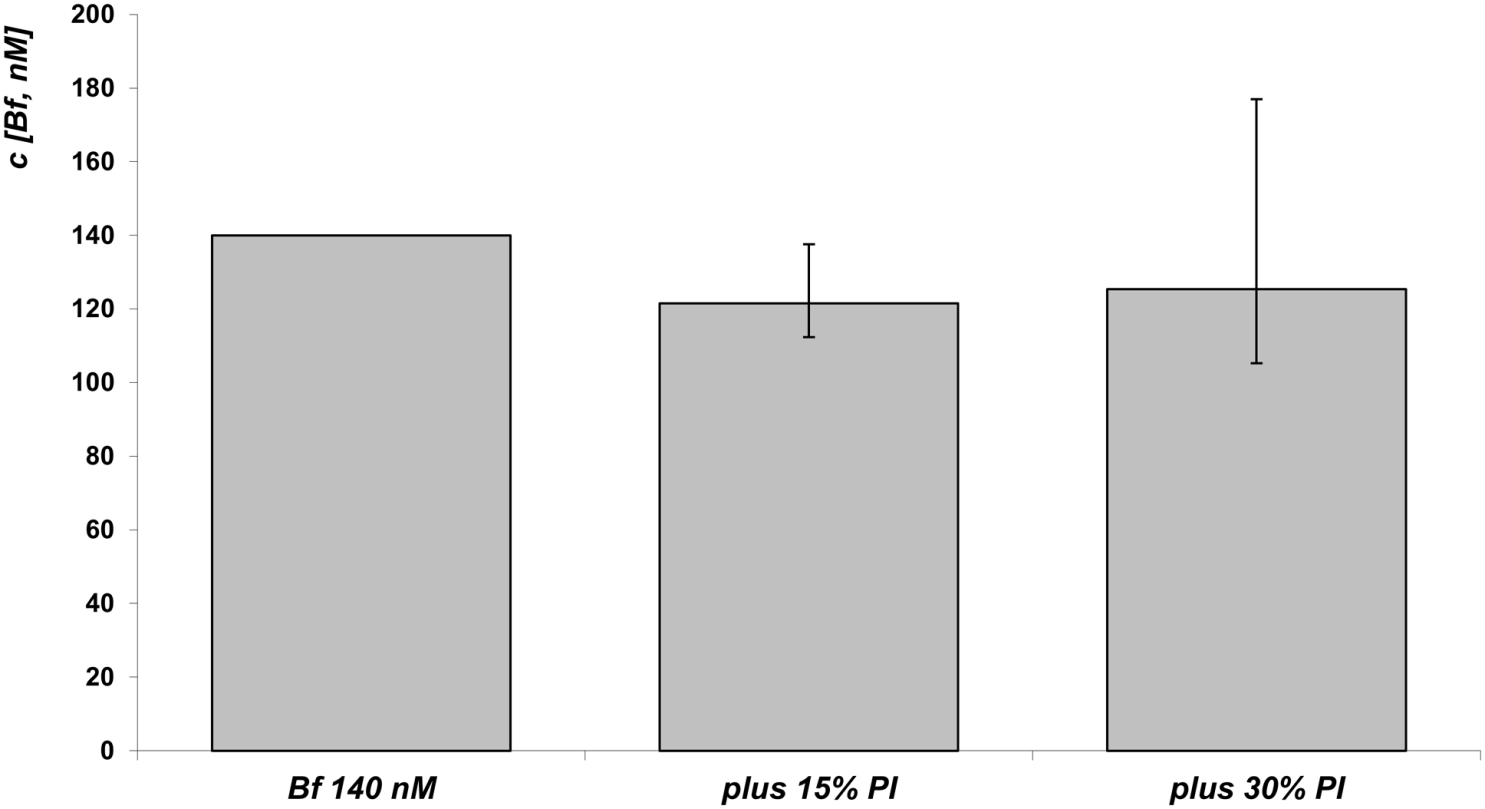
Effect of bilirubin PI on Bf concentrations.

To investigate whether bilirubin PI might affect Bf levels, we directly analyzed this effect by measuring Bf using a peroxidase method. The addition of 3.6 and 7.2 μM of bilirubin PI (corresponding to 15% and 30% of total bilirubin concentration, respectively) had no effect on the Bf concentration (Fig 4).

To a solution with 140 nM Bf concentration (approximately 24 μM bilirubin), bilirubin PI were added in increasing concentrations (15% and 30%); the resultant bar was constructed from the difference between the decrease of absorbance after addition of bilirubin PI or DMSO to the bilirubin solution. n = 6 for each group.

These data were further confirmed by the study of lumirubin-albumin binding (Table 2), where a fluorescence quenching method was used for the estimation of the albumin binding constant (*Ka*) for bilirubin and lumirubin. Lumirubin had a significantly lower *Ka* compared to bilirubin (Table 2). Its effect was comparable with that of biliverdin, which does not affect bilirubin binding to serum albumin because their high-affinity binding sites are located in two different subdomains (IB for biliverdin, and IIA for bilirubin). In line with this conclusion, the bilirubin-albumin binding constant was notably affected by gossypol, a displacer of bilirubin from HSA [31,32]. Therefore, lumirubin only moderately affected bilirubin binding to HSA (Table 2).

**Table 2:** The binding constant for the ligand-HSA complexes.

	bilirubin-HSA	lumirubin-HSA	bilirubin-(HSA-biliverdin)	bilirubin-(HSA- gossypol)	bilirubin-(HSA-lumirubin)
***Ka* [M**^**-1**^**]**	(1.8±0.3)·10^8^	(9.3±0.8)·10^5^	(1.1±0.4)·10^8^	(1.4±0.2)·10^5^	(8.7±1.3)·10^7^

HSA, human serum albumin; *Ka*, the binding constant

**Fig 5:**
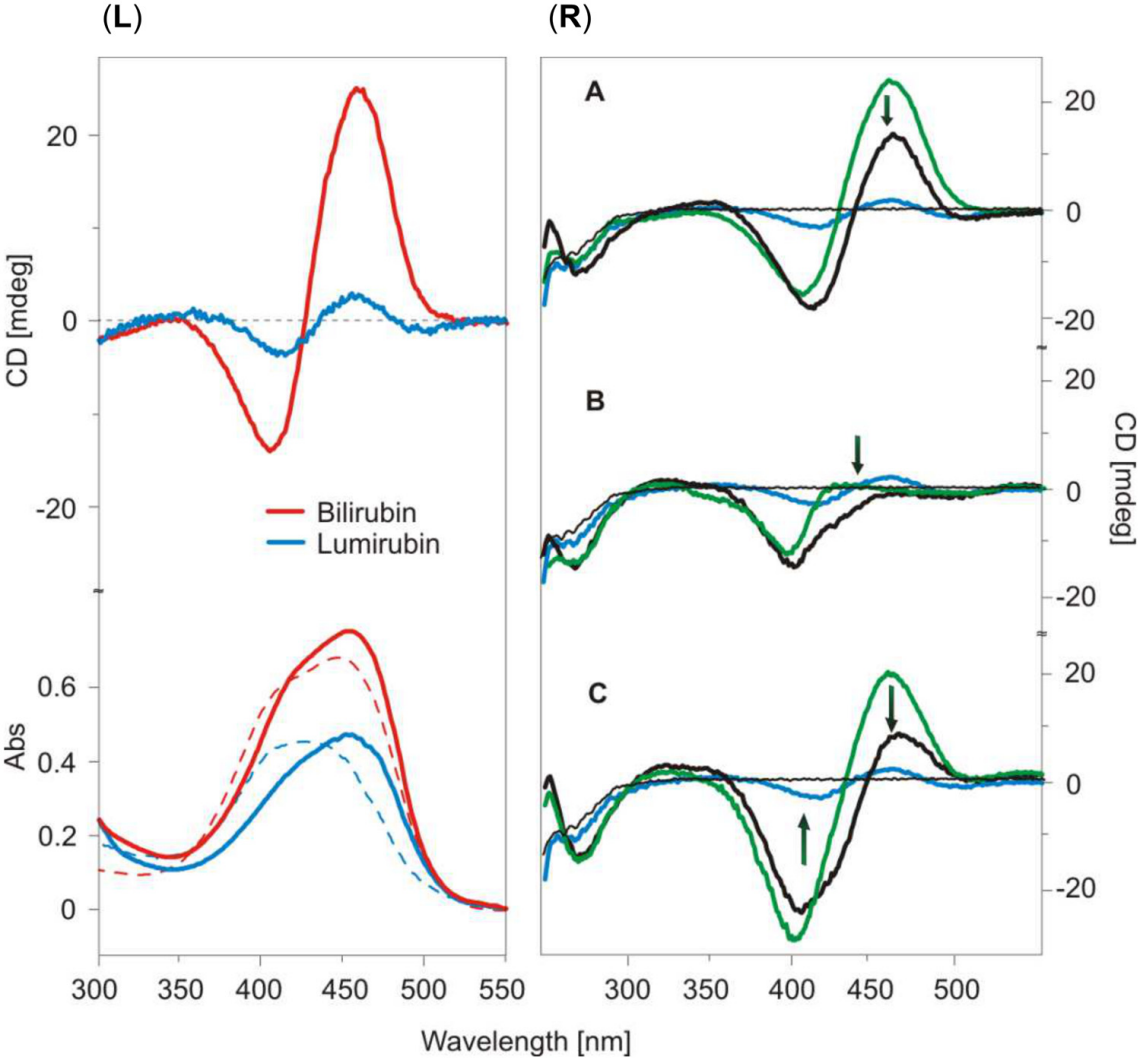
**(*Left panel*) CD and UV/Vis absorption spectra of unbound (broken line), lumirubin (blue), bilirubin (red), and their complexes with HSA (full line)**. Pigment/HSA molar ratio = 1/1; *c* (pigment) = 1.5 x 10^−5^ mol/L. Bilirubin was dissolved in 0.1 mol/L NaOH and mixed with the HSA solution in PBS (pH 7.4) at the molar ratio [pigment]/[HSA] = 1/1, the concentration of the pigment was 1.5 x 10^−5^ mol/L. **(*Right panel*) Effect of lumirubin on HSA binding with different marker ligands: bilirubin (A), hemin (B), and with both bilirubin and hemin (C)**. CD spectra of HSA-marker ligand are shown in green, lumirubin-HSA complex and lumirubin bound to the complex HSA-marker ligand are shown as blue and black full lines, respectively. Black arrows show the changes in signals after formation of lumirubin-(marker ligand-HSA) complexes. Lumirubin/HSA/marker molar ratio = 1/1/1.

The results of the CD analyses further supported this conclusion. Bilirubin was found to be bound to a high-affinity site inducing the CD positive couplet [460(+)/410(-) nm] that is characteristic for the bound P-conformer of bilirubin (see Fig 5, *left panel*). In the complexes with HSA, bilirubin shows an absorption maximum at 475 nm, which is shifted closer to the red spectrum compared to the unbound pigment in solution (440 nm; as can be seen on Fig 5, *left panel*). In contrast, CD spectrometric analysis of the lumirubin-albumin complex revealed that lumirubin has one binding site on HSA with right-helical conformation (it is also a P-conformer) of the bound pigment [weak positive couplet 445(+)/407(-) nm; Fig 5, *left panel*]. The obtained lumirubin-HSA complex had also a slight red-shifted absorption band with the maximum at 445 nm, compared to unbound lumirubin that absorbed at 430 nm.

To clarify the albumin domain that binds lumirubin, three different complexes of HSA and the marker ligand were used (Fig 5, *right panel*). The HSA-bilirubin system (green line, Fig 5A) was first compared with that of HSA-lumirubin (blue line). The resulted signal (black line) of the lumirubin-(HSA-bilirubin) complex is not the sum of lumirubin-HSA and bilirubin, and has indicating that the bilirubin and lumirubin binding sites are not independent. Lumirubin is bound close to the bilirubin high-affinity binding site, and it is able to affect bilirubin binding to HSA. In the HSA-hemin system (CD spectrum in green, Fig 5B), the addition of lumirubin did not induce the positive couplet characteristic for lumirubin bound to HSA (Fig 5B). It confirms that lumirubin and hemin bind to the same binding site. The binding site of lumirubin is localized in the subdomain IB [35] (or at a close distance to it), so the hemin presented there hindered the lumirubin binding. However, in the case where both bilirubin and hemin were bound to HSA (green spectrum in Fig 5C), the addition of lumirubin led to a moderate decrease of the bilirubin signal. This strongly suggests that the binding of lumirubin moderately affects bilirubin binding to HSA, and that the lumirubin binding site is localized close to the hemin and bilirubin binding sites.

Collectively, our data indicate that lumirubin only negligibly influences bilirubin binding to albumin. Although lumirubin binds to albumin, its binding has a much lower affinity and occurs at a different binding site. Nevertheless, the binding sites for bilirubin and lumirubin are at a close distance to one another, and these binding sites are not independent.

### The effect of bilirubin PI on SH-SY5Y cell viability

**Fig 6:**
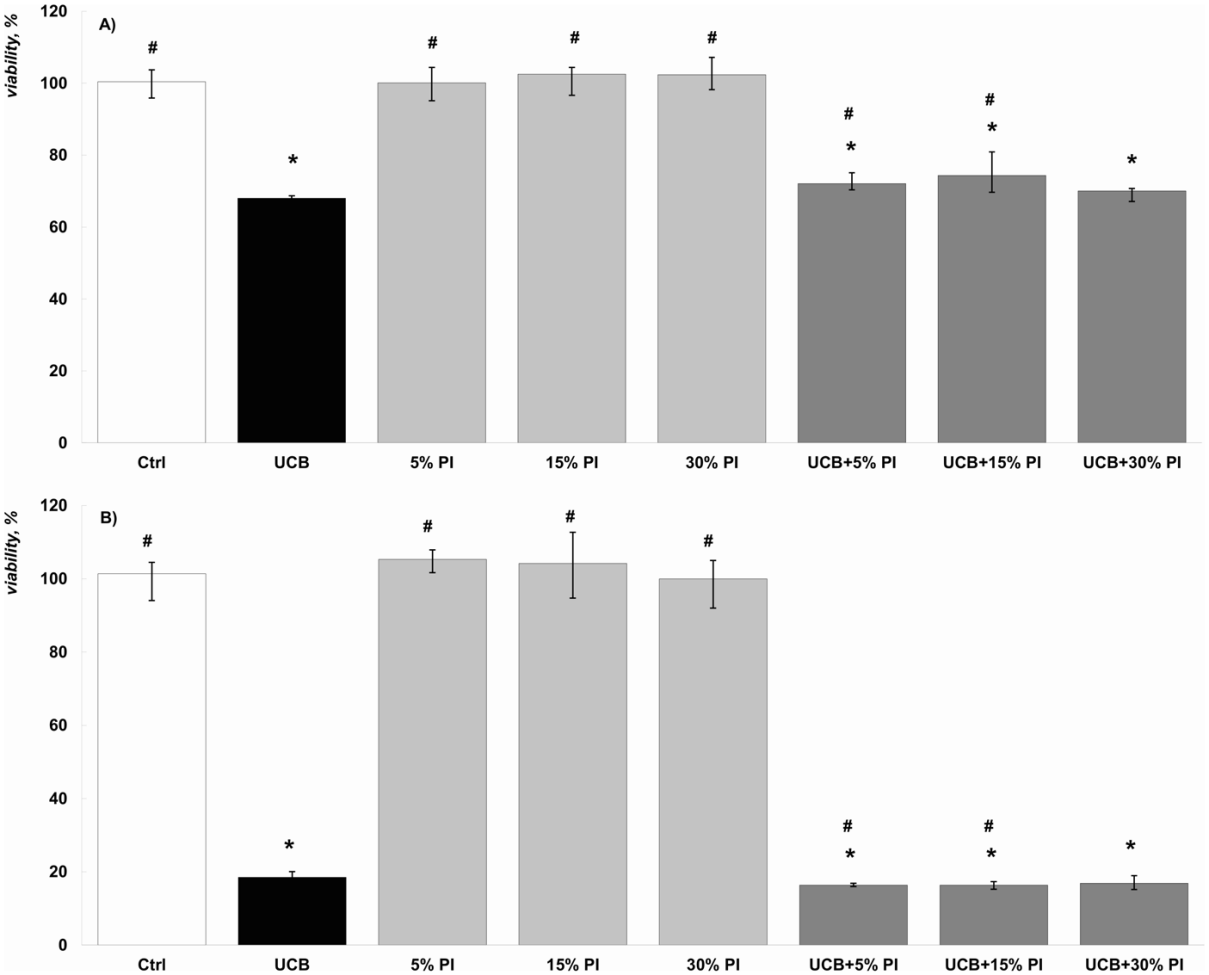
The effect of bilirubin and bilirubin PI on the cell viability in our *in vitro* model. (A) 24 hr exposure; n = 7. (B) 48 hr exposure; n = 4. * p < 0.05 vs. control; ^#^ p < 0.05 vs. bilirubin. Cell viability tested by CellTiter-Glo test; exactly the same results were obtained with MTT test. UCB, unconjugated bilirubin.

The short-term exposure (60 min) of bilirubin or its PI did not have any effect on cell viability (data not shown). However, cell viability was significantly reduced after the 24 or 48 hr cell treatments with bilirubin, and this effect further increased after 48 hours of exposure (Fig 6A and 6B, respectively). In contrast, bilirubin PI did not affect cell viability, even after 48 hr exposure and a high concentration used (30%) (Fig 6A and 6B).

### The effect of bilirubin and bilirubin PI on expression of genes involved in the heme catabolic pathway and cell cycle progression

Bilirubin is the final product of the heme catabolic pathway, and its formation is under the control of both HMOX and biliverdin reductase (BLVRA). Since these enzymes are protective tools of the organism against increased oxidative stress [41,42] (as well as responsible for bilirubin production), we investigated the effects of PI on the expression of these genes.

The expressions of both *HMOX1* and *HMOX2* were significantly increased after 1 hr of exposure to toxic concentrations of bilirubin, and the same trend was observed also for *BLVRA* (Table 3). These changes in mRNA expressions were, however, not translated onto increase of HMOX activity (data not shown). Interestingly, exposure to bilirubin PI did not lead to the changes in mRNA expression profiles seen after exposure to bilirubin.

**Table 3:** The effect of bilirubin and bilirubin PI on expression of selected genes.

	Bilirubin	15% PI	Bilirubin+15% PI
**HMOX1**	232.2 (197–240)*	105.6 (93–109)	277.1 (175–358)*
**HMOX2**	130.3 (129–166)*	84.8 (74–88)	100.3 (91–151)
**BLVRA**	142.9 (109–179)	86.3 (53–122)	158.4 (94–180)
**MRP1**	76.3 (71–90)	84.9 (51–100)	76.7 (61–89)
**MDR1**	54.9 (35–84)	86.3 (35–113)	87.3 (33–99)
**Cyclin D1**	71.8 (64–157)	64.5 (52–81)*	175.3 (137–232)*
**Cyclin E**	78.9 (50–126)	76.4 (59–84)	84.3 (77–106)

data are expressed as % of control (median and IQ range), n = 5 for each measurement. PI, bilirubin photoisomers

* p < 0.05 vs. control

Additionally, no difference was found in the expressions of either *MRP1* or *MDR1*—genes coding transporting proteins responsible for export of multiple compounds also possibly including bilirubin [43,44]. Expressions of genes encoding cyclins D1 and E involved in regulating the G_0_ to S phase, and G_1_ to S phase transition, respectively [45,46] tended to be down-regulated upon exposure to bilirubin and bilirubin PI (Table 3). Nevertheless, these mRNA expression changes were not functionally translated, as evidenced by flow cytometry analysis of the cell cycle phase of the SH-SY5Y cells exposed to bilirubin, bilirubin PI, or their mixture. This analysis revealed that compared to control cells, treatments with studied pigments had no effect on the cell cycle progression after either short or long exposure (data not shown).

## Discussion

Phototherapy is a non-invasive and effective treatment for neonatal hyperbilirubinemia, as well as one which facilitates the disposal of toxic bilirubin and avoids brain injury. Since its development in the 1950’s, it has become a standard and widely available treatment for this condition.

Phototherapy can reduce serum bilirubin by its conversion into its structural photoisomers and photooxidation products, which are excreted from the human body without the need of further biotransformation in the liver. It is generally believed that bilirubin photoisomers are non-toxic; however, no clear evidence for this viewpoint exists, and insufficient data about bilirubin PI's biological effects have thus far been provided. [16–23]. Of note is the fact that in none of these studies were the pure forms of the bilirubin photoderivatives used, most likely because of their complicated isolation and handling. To the contrary, short-term as well as long-term side effects of phototherapy have been repeatedly reported [7–12]; the mechanisms of which are unclear and might theoretically be accounted for by the biological activities of bilirubin PI.

In our experiments, we were able to successfully separate 18 different bilirubin photoderivatives generated during photo-irradiation of bilirubin solution. Out of these, we identified and isolated the major bilirubin photoderivatives (ZE/EZ-bilirubin and lumirubin) in sufficient purity and quantity for biological studies.

We were able to demonstrate that these bilirubin PI do not increase Bf levels, and thus do not increase bilirubin toxicity *per se*. Lumirubin was found to have only one binding site on HSA, and this binding site was the same as for hemin (i.e. in the subdomain IB or close to it). Nevertheless, the bilirubin and lumirubin binding sites on albumin are not totally independent, because the lumirubin binding site is close to the bilirubin high-affinity binding site; to a certain extent it is also able to lower bilirubin's ability to bind to HSA. However, this effect is probably not clinically relevant. In support of this data, we confirmed that lumirubin-HSA's binding constant is much lower, compared to bilirubin. These data collectively demonstrate that although lumirubin binds to albumin, the binding has no biological relevance in terms of any possible influence on bilirubin-albumin binding.

The experiments, focused on the potential cytotoxicity of major bilirubin PI, revealed no apparent effects on cell viability in our *in vitro* model, even after prolonged exposure. This was in striking contrast with the known toxic effects of bilirubin. This indicates that only unconjugated bilirubin is toxic, and that the conformational changes induced by irradiation almost completely abolishes the noxious effects of the pigment on the cell.

Neither bilirubin, nor bilirubin PI had any functional effect on two key enzymes (HMOX and BLVRA) important in heme degradation and bilirubin production. The lack of any effect of bilirubin PI on *BLVRA* mRNA expression was not expected, since biliverdin to some extent is also produced during phototherapy [6]. Similar negative results of bilirubin/bilirubin PI exposure were also found for *MRP1* and *MDR1* mRNA gene expression, indicating that these transporters are not inducible, at least in our cell system, by either bilirubin or their PI.

Our study has several limitations. First, we only assessed the biological effects of three major bilirubin PI, ZE/EZ-bilirubins and Z-lumirubin, and thus we cannot exclude that other photoproducts or bilirubin oxidation products formed during phototherapy, especially those short-lived and not detected in our study, might be toxic. Thus, it is still needed to be carefully assessed, whether minor oxidation products produced from bilirubin during phototherapy can exert any biological action. In addition, our studies were only performed on one cell line, representing a neuronal *in vitro* model. However, other brain cells, such as astrocytes, microglia, or even endothelial cells should also be tested; ideally in an organotypic brain slice *ex vivo* model to bring conclusive evidence.

In conclusion, our data indicate that the major bilirubin PI, ZE/EZ-bilirubin and lumirubin, seem to be biologically inert, and do not exert any negative biological effects. The side effects of phototherapy, theoretically attributable to bilirubin PI, are most likely due to other mechanisms.
